# Ascent of the conus medullaris in human foetuses: a systematic review and meta-analysis

**DOI:** 10.1038/s41598-022-15130-9

**Published:** 2022-07-25

**Authors:** Lucas Costa Almeida, Yasmin Juliany de Souza Figueiredo, André Pinheiro Zylberman, Diogo Costa Garção

**Affiliations:** Department of Morphology, Center for Biological and Health Sciences, Federal University of Sergipe, Av. Marechal Rondon, s/n - Jardim Rosa Elze, São Cristóvão, SE 49100-000 Brazil

**Keywords:** Anatomy, Medical research, Neurology

## Abstract

The aim of the present systematic review and meta-analysis was to identify when the ascent of the conus medullaris occurs in human foetuses considering differences in evaluation methods and sample characteristics. Five databases were searched for relevant articles using different combinations of keywords. Article selection and data extraction were performed independently by two reviewers. Disagreements were resolved by a third reviewer. The variables were distributed into four groups according to the gestational age of the specimens: I (13–18 weeks); II (19–25 weeks); III (26–32 weeks); IV (33 weeks to the probable date of birth). Eighteen articles were included. The majority used imaging exams as the evaluation method. Cadaveric dissections were reported in the remaining articles. Only morphological studies were included in the meta-analysis. Significant ascent occurs between groups I and III as well as groups II and IV. Despite the considerable heterogeneity among the studies included in the present review, the findings enabled the determination that the conus medullaris reaches its normal birth level by the 26th week. Further analyses should be performed based on nationality and ethnicity to diminish the heterogeneity of the data.

## Introduction

The spinal cord (SC) is a cylindrical structure of the central nervous system between the brainstem and the lower edge of the first lumbar vertebra. The SC receives peripheral information and controls voluntary muscles of the torso and limbs. Despite the segmentary organisation, there are no macroscopical limits between the segments of the SC. Structurally, the SC has cervical and lumbosacral enlargements, from which nerve roots originate and extend to the upper and lower limbs, respectively^1^.

Topographically, the SC resides inside the spinal canal but only occupies part of the length of the canal after birth. The *filum terminale* is a delicate strand of fibrous tissue that attaches the lowest part of the SC, denominated the conus medullaris, to the coccyx^2^.

**Figure 1:**
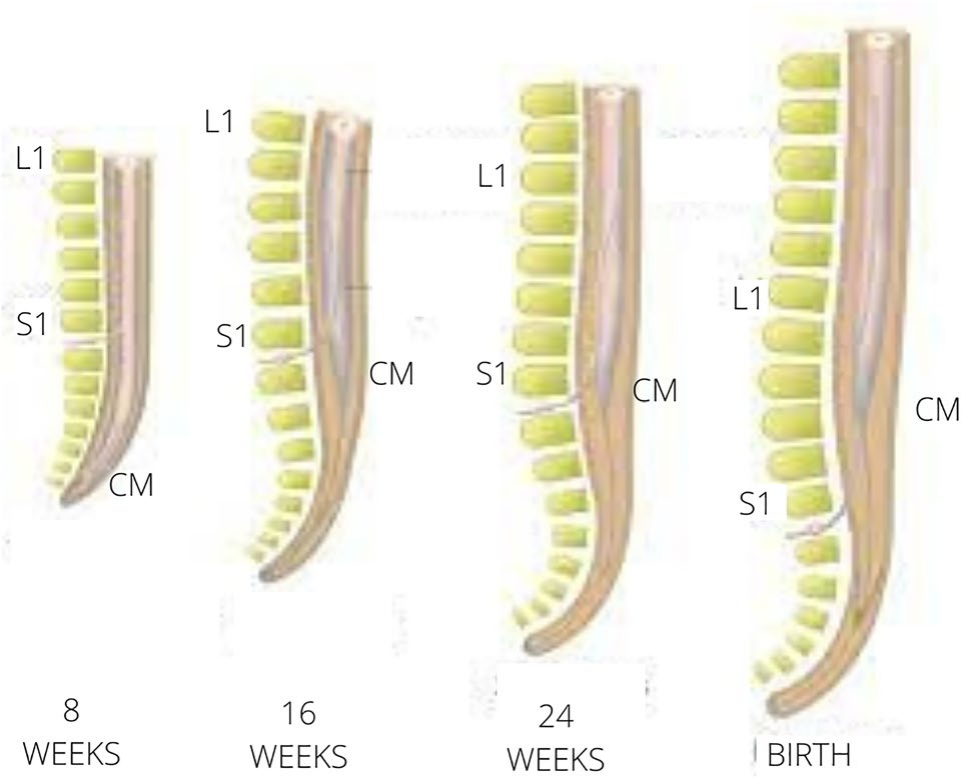
Ascent of the conus medullaris. Illustrations demonstrating progressive ascent of the conus medullaris (CM) during embryogenesis (8, 16 and 24 weeks) until birth and its relations to L1 and S1. Adapted from book “Cem milhões de neurônios”, Roberto Lent.

During foetal development, the conus medullaris (CM) ascends from a lower position in the spinal canal to its final location at birth (approximately at the level of the L2 vertebra)^3,4^ (Fig. 1). During intrauterine life, non-linear growth of the spinal cord occurs relative to the spinal canal, which is the main cause of the apparent ascent of the CM^1^.

The main methods reported in the literature for the evaluation of the position of the CM and its ascent are anatomical studies on post-mortem foetuses^5^ and imaging exams, such as sonography^6^ and magnetic resonance^7^. With ultrasound, the CM is identified as a dark triangular structure with two surrounding echogenic lines at the caudal portion of the SC^8,9^. The common methods for investigating the ascent of the CM are the determination of its position in relation to spinal level and the measurement of the distance between the CM and the last sacral vertebra^10^. Regarding both methods, it is important to highlight that flexion of the spine does not cause the conus medullaris to change position in fresh human cadavers, and it is also unlikely that the conus would change position during spine flexion during imaging^11^.

Knowledge of the normal growth pattern of the SC and its components can assist in understanding its development and birth defects. For example, a lower CM may indicate inadequate ascent, which raises suspicions of occult spinal dysraphism^12^. Several studies have reported that the determination of the location of the CM is valuable for the diagnosis of tethered cord syndrome. Prenatal diagnosis of this syndrome is important to determining the prognosis of affected foetuses. However, the detection of minor deformities of the foetal SC within the uterus remains a challenge^13^.

Information on the foetal CM can be used to guide to lumbar punctures, surgeries, and imaging studies of the neonate SC, especially when signs indicate tethered spinal cord or other anomalies. This is particularly important, as surgeries performed after the occurrence of symptoms have a low probability of correcting the neurological damage, but deterioration can be arrested if the procedure is successful^14,15^. Moreover, a dataset on normal CM development could be used as a reference for comparisons to potentially pathological conditions. However, the precise moment of CM ascent remains unknown, and the literature offers conflicting data, which hinders the establishment of a clear pattern^3^.

Therefore, the aim of the present systematic review and meta-analysis was to identify when the ascent of the conus medullaris occurs in human foetuses considering differences in evaluation methods and study samples. An additional objective was to determine whether there is a CM ascent pattern.

## Method

### Outline of study

This review was conducted in accordance with the guidelines specified in the Cochrane Collaboration Handbook, Version 5.2. The protocol for this systematic review was registered in the PROSPERO database (Registration code: CRD42020200839).

The research question was formulated using the POT acronym (Population–Outcome–Type of study), the aim of which is to systematise and optimise the search process in databases. Using the established criteria (P, human foetuses; O, ascent of the CM; and T, cross-sectional anatomical studies), the following question was posed: When does the ascent of the CM occur in human foetuses?

### Search strategy

Five online databases were searched for articles meeting the pre-established criteria: National Library of Medicine (MEDLINE-PubMed), SciELO, Web of Science, Lilacs, and Scopus. Different combinations of the following keywords were used by two independent researchers: “evaluation”, “conus medullaris”, “position”, “development”, “spinal cord”, “human foetus”, “distance”, and “anatomy”. After the retrieval of relevant references, the titles were analysed and duplicates were removed.

### Study selection

Two reviewers performed the article selection process in two stages: analysis of abstracts for potentially eligible articles and full-text analysis. Divergences of opinion were resolved by consulting a third impartial reviewer. The following were the inclusion criteria: studies on human foetuses; gestational ages between 13 and 40 weeks; the use of appropriate methods to locate the CM, such as magnetic resonance, dissection, or ultrasound. Studies that only used foetuses with brain or spine abnormalities, systematic reviews, case reports, and meta-analyses were excluded. Papers from the twentieth century were also included, as the dissection methodology was only used in this period.

### Data extraction

Two reviewers performed the data extraction independently, with the participation of a third impartial reviewer to resolve disagreements, when needed. The following data were collected: main author’s name, publication date, duration of the study, nationality of the specimens, analysis method, number of foetuses, proportion between males and females, number of foetuses by gestational age, location of the CM in the spine, formula for comparing gestational age and CM distance to a reference measure, formula for comparing femur size and CM distance to a reference measure, and measures taken to diminish intra-observer and inter-observer variability.

The methodological quality (risk of bias) of the studies was appraised using the Anatomical Quality Assurance (AQUA) tool designed by the International Evidence-Based Anatomy (iEBA) working group^16^. The AQUA tool probes for risk of bias in five domains (objectives and subject characteristics,study design; characteristics of methods; descriptive anatomy; and reporting of results). The risk of bias for each domain is classified as ‘high’, ‘low’ or ‘unclear’.

### Meta-analysis

The L2 level was established as a parameter, as it is approximately the final location of the CM at birth^3,4,6,17^. Therefore, meta-analysis was conducted by gestational age to determine the frequency of the CM at or above L2. To compare the results, the distribution of the data (normal or non-normal) was determined using the Kolmogorov-Smirnoff test, followed by the Kruskal–Wallis test with Dunn’s post-hoc test (*p* < 0.05).

### Statistical analysis

To improve the evaluation and unify heterogeneous data, the variables were distributed into four groups according to the gestational age of the specimens: I (13–18 weeks); II (19–25 weeks); III (26–32 weeks); IV (33 weeks to the probable date of birth). In one study, a gestational age of 40 weeks was established as birth.

Considering this division, every specimen received a score from 1 to 5 depending on the location of the CM in the lumbar spine: 1—L5; 2—L4; 3—L3; 4—L2; 5—L1. For foetuses with a CM in an intervertebral position, the lower vertebra was considered. Using these data, the Kruskal–Wallis test was performed followed by Dunn’s post hoc test (*p* < 0.05) to determine the occurrence of significant differences among groups. Pearson’s correlation test (*p* < 0.05) was also used to determine the occurrence of correlations between CM location and gestational age.

## Results

### Article selection

**Figure 2:**
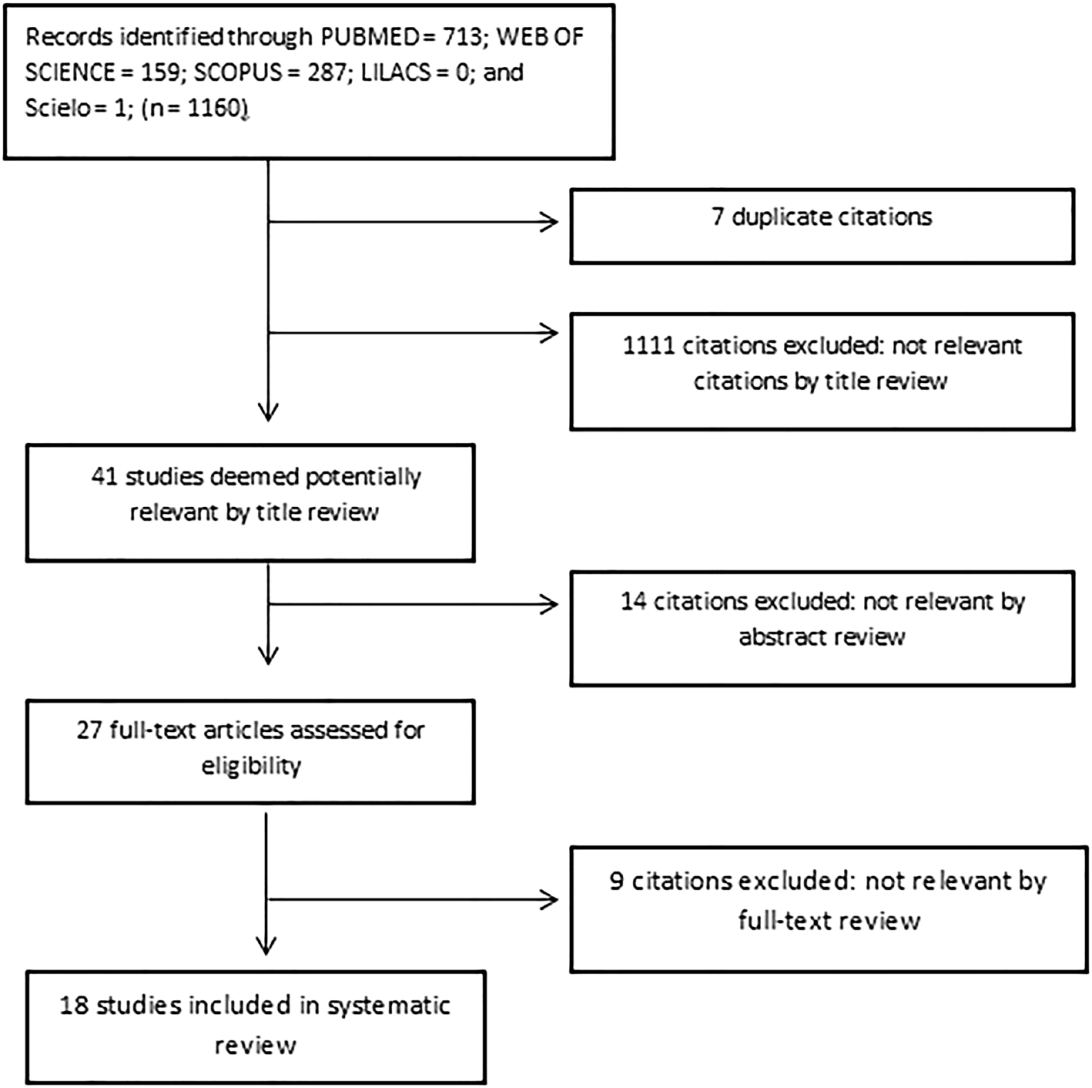
Flowchart of article selection process in accordance with PRISMA guidelines.

The search led to the retrieval of 1160 potentially relevant studies. After the removal of duplicates and screening of the titles, 41 abstracts were submitted to analysis, leading to the exclusion of 14 articles for not mentioning the position of the CM or involving abnormal foetuses (Table 1). After the full-text analyses, nine articles were excluded for not meeting inclusion criteria, mainly due to studying new-borns rather than foetuses. At the end of the selection process, 18 articles were included in the present systematic review and meta-analysis (Fig. 2).

**Table 1:** Reasons for excluding the articles from further analysis.

Study	Nationality	Reason for exclusion
Kawahara et al.^19^	Japan	Data from newborns rather than foetuses
Wilson and Prince^20^	USA	Data from infants rather than foetuses
Filly et al.^21^	USA	No CM position data
Wolf et al.^22^	Germany	Data from newborns rather than foetuses
Beek et al.^23^	Netherlands	Data from newborns rather than foetuses
Cochrane et al.^24^	Canada	Data from abnormal foetuses only
Nolting et al.^25^	Denmark	No CM position data
Duczkowska et al.^26^	Poland	No CM position data
Blondiaux et al.^27^	France	No CM position data
Thakur and Lowe^28^	USA	Data from infants rather than foetuses
Mottet et al.^29^	France	Data from abnormal foetuses only
Nagaraj et al.^30^	USA	Data from abnormal foetuses only
Miao et al. ^12^	China	No CM position data
He et al. ^13^	China	No CM position data

### Characteristics of studies included

Among the 18 studies (n = 3189 useful foetuses), 15 used imaging exams as the evaluation method: sonography (12 studies; 2752 useful foetuses; 66.7%) and magnetic resonance (three studies; 158 useful foetuses; 16.7%). The remaining studies involved visual analyses during cadaveric dissections (three studies; 279 useful foetuses; 16.7%). Eleven of the studies (61.1%) performed morphological analyses, comparing the CM to its corresponding vertebral level. Six studies (33.3%) performed morphometric analyses, measuring the distance between the CM and a selected position of the sacrum. One study (5.6%) used both types of analysis. Only morphological results were considered in the meta-analysis.

The largest number of studies were from Europe (seven studies; 1525 useful foetuses; 47.8%), followed by Asia (six studies; 1414 useful foetuses; 44.3%), the Middle East (three studies; 213 useful foetuses; 6.8%), South America (one study; 24 useful foetuses; 0.7%), and North America (one study; 13 useful foetuses; 0.4%). Only nine articles mentioned the study period (range: three months to seven years). The years of publication ranged from 1970 to 2019 (Table 2).

**Table 2:** Characteristics of studies included in present review.

Studie	Studie	Analysis method	Assessment tool	Useful sample	GA (weeks)
Barson^31^	England	Morphological	*Post mortem*	189	13-Birth
Vettivel^33^	india	Morphological	*Post mortem*	65	10–24
Robbin et al.^14^	USA	Morphological	Ultrasound	13	19–36
Malas et al.^8^	Turkey	Morphological	*Post mortem*	25	14–19
Zalel et al.^3^	Israel	Morphological	Ultrasound	110	13-Birth
Perlitz et al.^40^	Israel	Morphological	Ultrasound	78	20–24
Hoopmann et al.^9^	Germany	Morphometric	Ultrasound	254	15-Birth
Arthurs et al.^4^	UK	Morphological	MRI	84	14–39
Huang et al.^7^	Taiwan	Morphological	MRI	50	20–38
Lei et al.^42^	China	Morphological	Ultrasound	145	20–38
Rodríguez et al.^36^	Spain	Morphometric	Ultrasound	101	20–32
He et al.^37^	China	Morphometric	Ultrasound	511	14-Birth
Rodríguez et al.^38^	Spain	Morphometric	Ultrasound	573	20–35
Mottet et al.^39^	France	Morphometric	Ultrasound	164	17–37
Zhao et al.^35^	Austria	Morphometric	Ultrasound	160	20–28
Yang et al.^43^	China	Morphological	Ultrasound	122	20–30
Zhai et al.^10^	China	Morphological e morphometric	Ultrasound	521	20–28
Manzone et al.^28^	Argentina	Morphological	MRI	24	13–22

GA: Gestational age.

### Appraisal of methodological quality

The AQUA tool revealed that two studies (11.1%) had a high risk of bias in the first domain (objective and subject characteristics) and nine (50.0%) had a high risk in the third domain (characteristics of methods). This was mainly due to the lack of demographic data presented in the studies and the omission of the researchers' experience and measures taken to lower intra-observer and inter-observer variability. All papers had a low risk of bias regarding the second, fourth, and fifth domains (study design, descriptive anatomy, and reporting of results, respectively) (Table 3).

**Table 3:** Results of appraisal of methodological quality using AQUA tool.

Studie	Domains				
	1	2	3	4	5
Barson^31^	−	−	−	−	−
Vettivel^33^	−	−	+	−	−
Robbin et al.^14^	+	−	+	−	−
Malas et al.^8^	−	−	+	−	−
Zalel et al.^3^	−	−	+	−	−
Perlitz et al.^40^	−	−	+	−	−
Hoopmann et al.^9^	−	−	+	−	−
Arthurs et al.^4^	−	−	−	−	−
Huang et al.^7^	+	−	+	−	−
Lei et al.^42^	−	−	−	−	−
Rodríguez et al.^36^	−	−	−	−	−
He et al.^37^	−	−	−	−	−
Rodríguez et al.^38^	−	−	−	−	−
Mottet et al.^39^	−	−	−	−	−
Zhao et al.^35^	−	−	−	−	−
Yang et al.^43^	−	−	−	−	−
Zhai et al.^10^	−	−	+	−	−
Manzone et al.^32^	−	−	+	−	−

+ (high risk of bias); − (low ris of bias).

### Primary outcome

**Figure 3:**
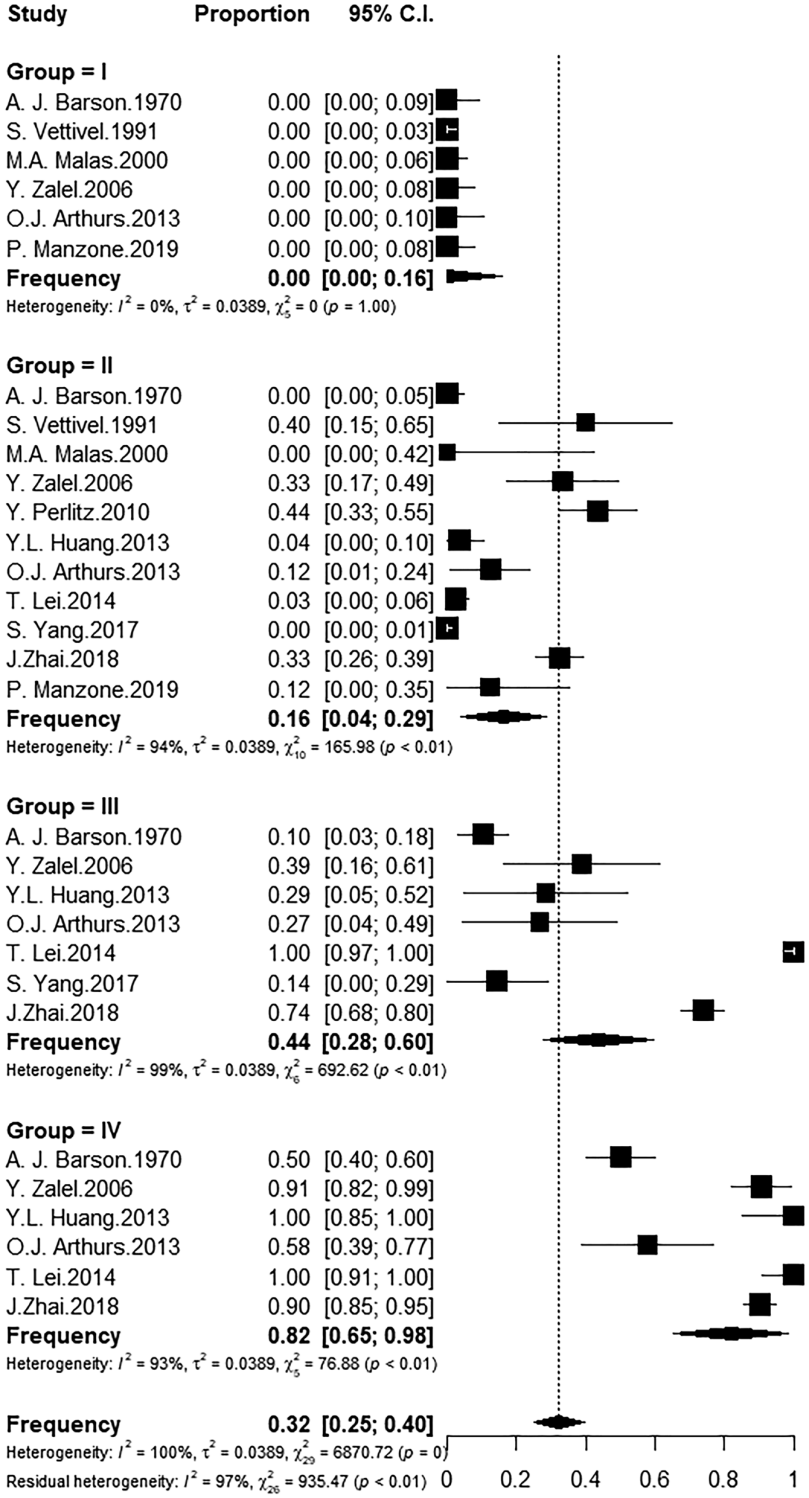
Frequency of foetuses with CM at L2 or above by gestational age group.

Among the 12 studies with a morphological analysis, one (8.3%^14^) was not included in the meta-analysis for not reporting the age of the foetuses. All foetuses in Group I (15.91 ± 0.14; 128 useful foetuses) had a CM lower than vertebral level L2. In Group II (22.27 ± 0.07; 577 useful foetuses), the frequency of the CM at L2 or above was 0.16, with a high rate of heterogeneity among the studies (I^2^ = 80%). This figure increased to 0.42 (I^2^ = 93%) in the seven studies that used foetuses in Group III (29.12 ± 0.10; 379 useful foetuses) and 0.82 (I^2^ = 91%) in the six studies that used foetuses in Group IV (35.76 ± 0.12; 344 useful foetuses) (Fig. 3). Thus, a statistically significant ascent of the CM occurred between Groups I and III as well as between Groups II and IV considering the frequency at L2 or above (*p* < 0.05, Kruskal–Wallis test and Dunn’s post hoc test). This analysis demonstrates that the foetal CM begins to reach vertebral level L2 in the 26th week, but a high rate of variability was found among the studies.

**Figure 4:**
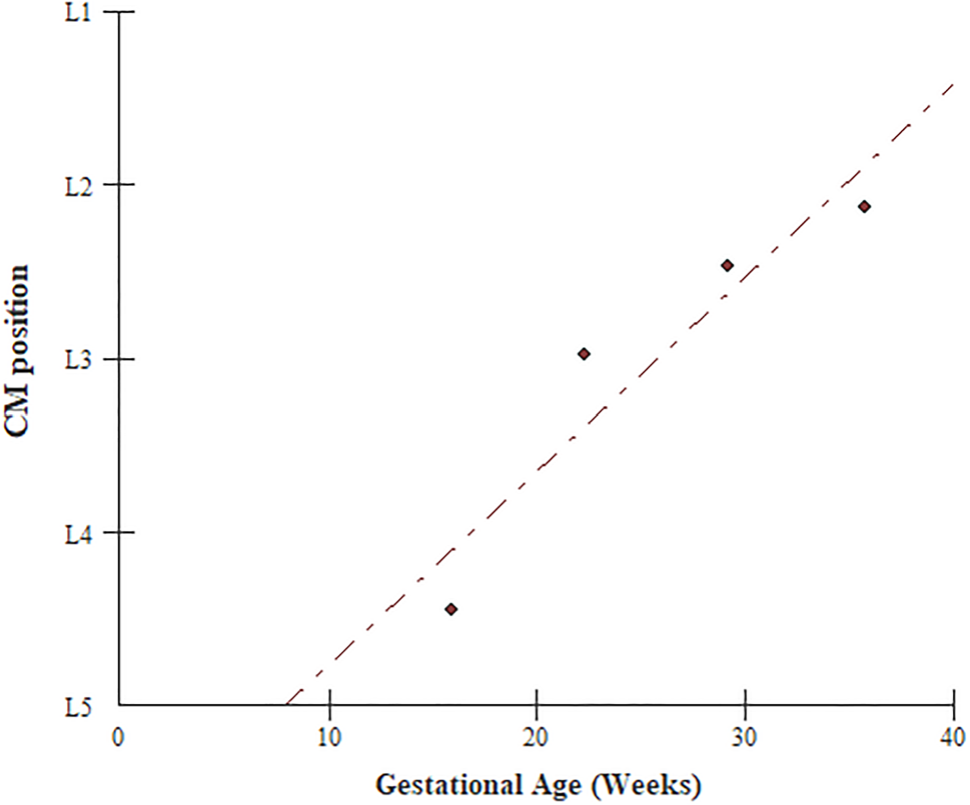
Correlation between gestational age and CM position.

Significant differences were found regarding the average vertebral level (L1–L5) that the CM reached per group (*p* < 0.05, Kruskal–Wallis test and Dunn’s post hoc test). This indicates that the ascent of the conus medullaris occurs throughout the entire period considered in this meta-analysis (from 13 weeks to the probable date of birth). Moreover, a strong positive correlation (r = 0.86; *p* < 0.05) was found between the average age in the four groups and the average position of the CM on each group (Fig. 4), demonstrating that the ascent process is directly correlated with gestational age.

Considering the seven studies with morphometric analysis (n = 2284 useful foetuses), five (71.4%) measured the distance between the CM and the last sacral centre of ossification (CS distance) and two (28.6%) measured the distance between the CM and S1 (CS1 distance).

### Secondary outcome

Considering only the morphological studies, the CM was lower than the L2 level in all foetuses in Group I, independently of the evaluation tool (anatomic dissection, sonography, or magnetic resonance). In the studies involving in post-mortem foetuses in Group II, the frequency of the CM at L2 or above was 0.05 (I^2^ = 79%), whereas this figure was higher in Groups III and IV (0.10 and 0.50, respectively). Only one study^18^ had useful foetuses in these two intervals.

**Figure 5:**
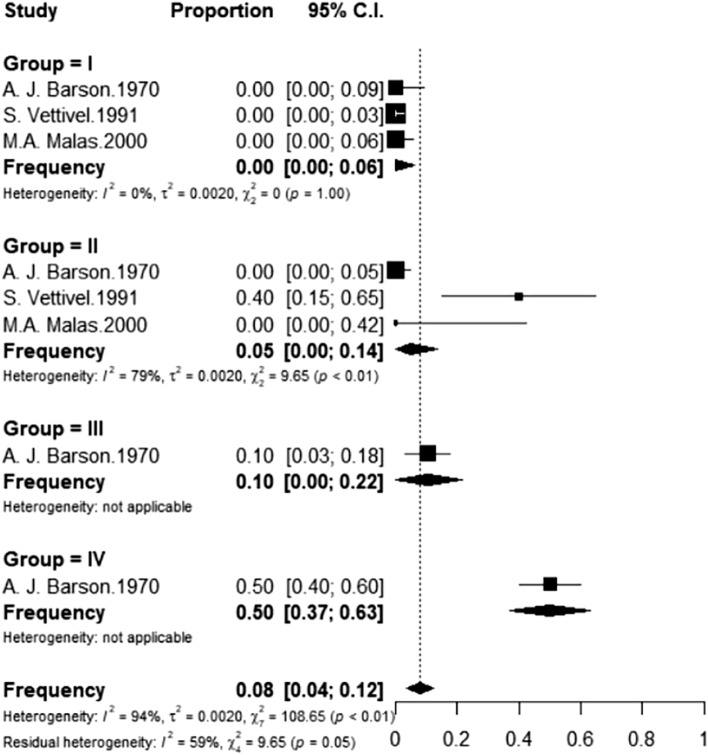
Frequency of foetuses evaluated post-mortem with CM at L2 or above distributed by gestational age group. Group I (13–18 weeks); II (19–25 weeks); III (26–32 weeks); IV (33 weeks to the probable date of birth).

**Figure 6:**
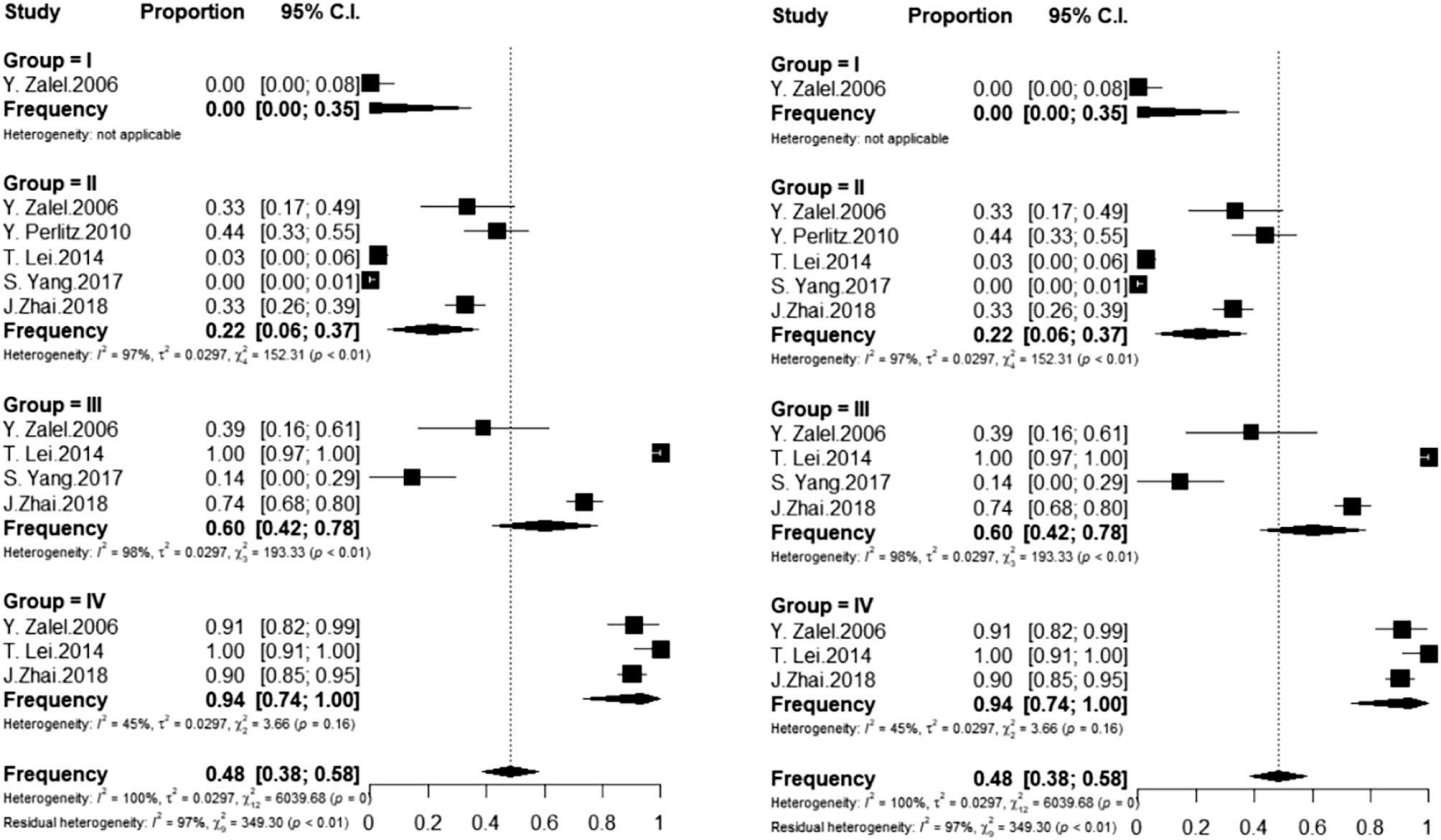
Frequency of foetuses evaluated by ultrasound with CM at L2 or above distributed by gestational age group. Group I (13–18 weeks); II (19–25 weeks); III (26–32 weeks); IV (33 weeks to the probable date of birth).

**Figure 7:**
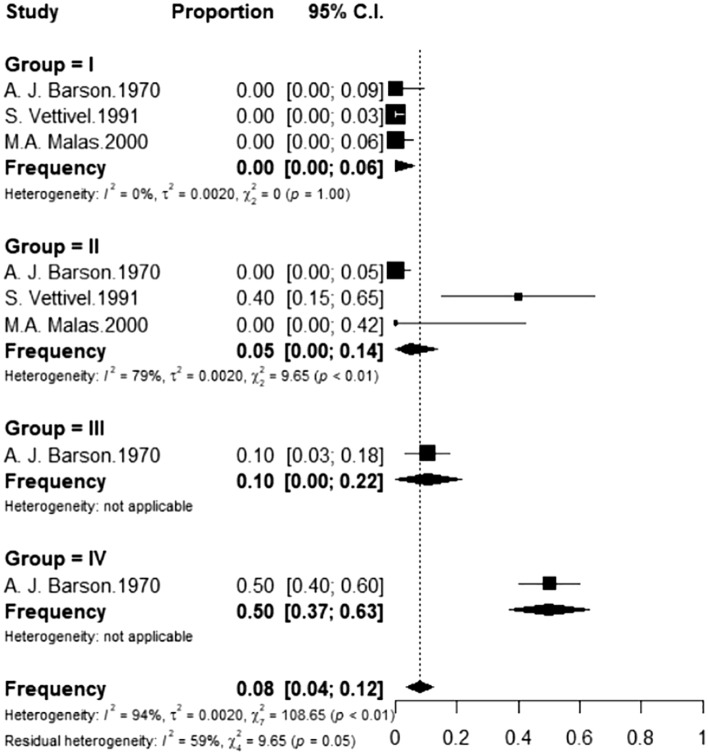
Frequency of foetuses evaluated by MRI with CM at L2 or above distributed by gestational age group. Group I (13–18 weeks); II (19–25 weeks); III (26–32 weeks); IV (33 weeks to the probable date of birth).

Among the morphological studies that employed ultrasound, the frequency in Groups II and III was respectively 0.22 (I^2^ = 97%) and 0.60 (I^2^ = 98%), whereas this figure increased to 0.94 in Group IV (I^2^ = 45%). In the studies that employed magnetic resonance, the frequency in Groups II and III was respectively 0.08 (I^2^ = 1%) and 0.28 (I^2^ = 0%), whereas this figure was 0.82 in Group IV (I^2^ = 91%) (Figs. 5, 6 and 7).

The variables addressed in the morphometric studies were gestational age (seven; 100%), femur size (six; 85.7%), biparietal diameter (three; 42.9%), abdominal circumference (three; 42.9%), head circumference (two; 28.6%), and foetal position (one; 14.3%). The studies analysed the data using linear regressions, which showed significant associations between these variables and the measurement of choice (CS distance or CS1 distance).

Only four of the studies included in the present review (22.2%) compared the ascent of the conus medullaris in male and female foetuses and none found a significant difference between sexes.

## Discussion

The studies included in the present systematic review revealed different CM ascension patterns. Barson^31^ suggest early ascension at 35 weeks, justified by slight disparity in the development of the SC when compared to the spine, with unequal growth patterns in the lumbar portion in relation to the other axial segments.

Manzone et al.^32^ analyzed the growth pace of the spine and SC, reporting steady, dissimilar values. The asymmetry between the SC level and spine is due to the disproportional growth of the two structures, which results in the ascent of the CM from S1 to L2 in the second trimester of pregnancy. These results were evident in this systematic review, which showed that the CM begins to appear above L2 between 19 and 25 weeks of pregnancy, with a progressively increasing frequency accompanying the increase in gestational age. This occurs after the secondary neurulation process, more specifically, after the retrogressive differentiation that occurs during the 11th week of gestation. The apparent ascent of the CM is progressively increasing in level, and starts to reach L2 from the 19th week in some cases, as it was evidenced in this study.

The present systematic review and meta-analysis showed that the CM reached level L2 or higher starting in the 26th week in most studies. However, some studies reported an earlier ascent, such as the one conducted in India by Vettivel^33^ using the anatomical dissection of post-mortem foetuses as the evaluation method, in which the CM was at level L2 or above in half the samples with a gestational age of 19–25 weeks. Recognizing the normal ascension pattern of the CM and identifying possible changes in the development of the SC can contribute to more accurate diagnoses and early interventions regarding syndromes such as anchored cord. It also could reduce the chances of a patient developing dysfunctions resulting from stretching and subsequent micro-impacts in the lumbar and sacral region.

The present results show that the CM ascension process occurs progressively through to the last weeks of pregnancy. In agreement with this finding, a study with magnetic resonance on post-mortem foetuses conducted in the United Kingdom found additional CM ascension in the third trimester of pregnancy^4^. In contrast, a study conducted in China involving morphological and morphometric analyses on a sample of 521 healthy foetuses of both sexes used ultrasound to measure the CM-S1 distance. The authors found that the foetuses began to present the CM at L2 in the 22nd week and that the CM was at or above L3 in the entire sample after 23 weeks. The CM was above L2–L3 after 32 weeks and above L2 in most foetuses with a gestational age of 37 weeks^10^.The disparity between the pattern of CM ascension between the studies could be associated with the ethnicity of the sample. However, more studies in different nationalities are needed to consolidate this conclusion.

Based on the correlation between average vertebral level and the CM, the present review revealed that the ascension of this structure occurs throughout the entire period considered, with sharp increase between the 10th and 25th weeks of pregnancy. During this period, the CM leaves the L5 level and reaches L3 in most cases, ascending progressively up to L2 or higher from the 26th week until birth.

Regarding morphology, the three studies^35,37,38^ that addressed the distance between the CM and the end of the sacrum performed analyses according to gestational age group. In the studies that addressed the CM-S distance, only one^10^ distributed the sample by gestational age group, while the other articles^9,36,39^, did not describe any division of the samples considering foetal age. In the present systematic review, all studies found a significant correlation between CS distance and gestational age. Thus, the disparity between spine development and the SC during weeks of gestation justifies the ascension of the CM. This confirms the strong positive correlations between the average age in the four groups and the location of the CM in each.

The studies that used post-mortem foetuses with either dissection^8,18,31^ or post-mortem magnetic resonance^4,32^ presented similar, low frequencies of foetuses with the CM at L2 from 19 to 25 weeks. In contrast, studies involving ultrasound performed on live foetuses^3,10,40^ found that the CM was at L2 in approximately half of the entire sample of foetuses with gestational age from 19 to 25 weeks. There are many reasons why post-mortem studies can present dissimilar results. The state and time of foetus conservation may influence the position of the SC. This may occur due to a postural change in vertebral kyphosis influenced by an element of non-physiological severity that diminishes the curvature and, consequently, reduces the CM level.

Furthermore, an Indian study with a post-mortem sample^41^ that evaluated the location of the CM through anatomical dissection reported levels above L2 in foetuses with a gestational age of 19–25 weeks. This divergence may be explained by the influence of ethnicity. The study reports that foetuses in southern India had a tendency toward higher CM ascension compared to those in northern India, consequently reporting a higher frequency of foetuses at or above L2 at a lower gestational age compared to data from other post-mortem studies. Vettivel^33^ confirmed this hypothesis, demonstrating a tendency toward positioning up to one vertebra above the average in gestational age groups among foetuses in southern India compared to those in northern India. Thus, the high variability in the position of the CM throughout the weeks of pregnancy weeks found in the present systematic review and meta-analysis could be influenced by the inclusion of studies with different ethnicities.

Regarding the morphometric approach, Hoopman et al.^9^ standardised this analysis using anatomical landmarks, such as the distance between the most caudal portion of the CM and the last ventral ossification center of the sacrum (CS1). To identify S1, the studies evaluated lumbosacral curvature using ultrasound. This approach was performed with the use of two tangents drawn at the last lumbar and sacral ossification nuclei from bottom to top, the intersection of which was the CM^10,35–39^.

Besides the morphometric method, the use of formulas to predict the normal CM ascension pace enables a more reliable assessment of SC development^42^. The seven morphometric studies included in this systematic review conducted linear regressions and found associations between the progressive ascension of the CM and gestational age, femur length, biparietal diameter, and BMI. Therefore, the routine quantification of the CS1 distance in imaging exams is viable. An early assessment could help diagnose spinal dysraphism, tethered SC, and other problems related to SC development. Prior detection is important to prepare parents for the anomaly and enable physicians to develop a surgical repair strategy in a timely manner to avoid irreversible neurological damage.

Regarding sexual dimorphism, despite the small number of studies that addressed this variable, no significant association was found among the articles included in the present review. Manzone et al.^32^ found no statistically significant difference in SC length between male and female foetuses in an Argentinian study performed with magnetic resonance. Perlitz et al.^40^ used ultrasound to analyse Israeli foetuses and also found no difference in CM position between the sexes.

## Study limitations

The main limitation of the present systematic review and meta-analysis is the high level of heterogeneity, which may be explained by the unequal distribution of studies among different countries. The lack of studies including different ethnicities makes it difficult to establish a global pattern of CM ascent. Moreover, the different methods of viewing the CM and assessing the CM level can exert an influence on the analysis and contribute to the highly heterogeneous data.

## Conclusion

This systematic review and meta-analysis concluded that the CM reaches the birth level (L2) in the 26th week of pregnancy. This information can be used to compare potentially anomalous cases to the clinically normal development of the CM. However, due to the ethnic differences, the different methods of viewing the CM, and the different methods of assessing CM level, the dataset was highly heterogeneous. Further analyses should be performed according to nationality and ethnicity to enable the description of possible CM ascension pattern by ethnic group and decrease the degree of heterogeneity in currently available data.
